# Using SPM 12’s Second-Level Bayesian Inference Procedure for fMRI Analysis: Practical Guidelines for End Users

**DOI:** 10.3389/fninf.2018.00001

**Published:** 2018-02-02

**Authors:** Hyemin Han, Joonsuk Park

**Affiliations:** ^1^Educational Psychology Program, University of Alabama, Tuscaloosa, AL, United States; ^2^Department of Psychology, The Ohio State University, Columbus OH, United States

**Keywords:** Bayesian statistics, Bayes factor, SPM, fMRI, second-level analysis, threshold

## Abstract

Recent debates about the conventional traditional threshold used in the fields of neuroscience and psychology, namely *P* < 0.05, have spurred researchers to consider alternative ways to analyze fMRI data. A group of methodologists and statisticians have considered Bayesian inference as a candidate methodology. However, few previous studies have attempted to provide end users of fMRI analysis tools, such as SPM 12, with practical guidelines about how to conduct Bayesian inference. In the present study, we aim to demonstrate how to utilize Bayesian inference, Bayesian second-level inference in particular, implemented in SPM 12 by analyzing fMRI data available to public via NeuroVault. In addition, to help end users understand how Bayesian inference actually works in SPM 12, we examine outcomes from Bayesian second-level inference implemented in SPM 12 by comparing them with those from classical second-level inference. Finally, we provide practical guidelines about how to set the parameters for Bayesian inference and how to interpret the results, such as Bayes factors, from the inference. We also discuss the practical and philosophical benefits of Bayesian inference and directions for future research.

## Introduction

The problem of widespread false-positive findings in the literature is drawing significant attention in scientific fields such as psychology, biology, and medicine (Ioannidis, 2005; Simmons et al., 2011; Pashler and Wagenmakers, 2012; Open Science Collaboration, 2015). Combined with other factors such as publication bias (Francis, 2012) and underpowered studies (Maxwell, 2004), false positives contribute to a more general problem, ‘reproducibility crisis,’ where an undesirably high proportion of published scientific results fails to be successfully replicated or reproduced when conducted again (Peng, 2015; Baker, 2016). Because the reproducibility crisis significantly undermines the reliability of science, it must be addressed for science to be credible.

Social and cognitive neuroscience is by no means an exception. False positives have been a subject of major concern in the field, especially when it comes to the analysis of fMRI data. Due to the extremely large number of tested hypotheses during fMRI data analysis, it is well known that the rate of false positives, or type I errors, can be extremely high when inappropriately dealt with (Bennett et al., 2009). As a result, the prevention of false positives has been of great interest among neuroscientists. For example, multiple comparison procedures such as Bonferroni correction and controlling for False Discovery Rates (FDRs) have been employed in practice to counter the problem (Benjamini and Hochberg, 1995). These procedures are currently implemented in popular fMRI data analysis software so that researchers can use them in their analyses.

Unfortunately, reports suggest that such may not be working well in practice. For example, an analysis by Han and Glenn’s (2017) shows Bonferroni correction and FDR are inappropriate for use because they are either too harsh or liberal in controlling for type I errors. Instead, they found that random field theory (RFT) familywise error correction (FWE)-applied voxel-wise thresholding struck a balance between the two methods, and they deemed it acceptable for fMRI data analysis in moral psychology.

Another recent report showcases an extremity where popular methods for controlling for type I error fail miserably (Eklund et al., 2016). Their analysis showed that, in the case of cluster-wise inferences using RTF, the analytic procedures employed in fMRI data analysis software, such as SPM, FSL, and AFNI, did not attain the claimed nominal significance levels. Instead, they exhibited greatly inflated false positive rates, sometimes as high as 70%, which is clearly undesirable. These observations are alarming in that published results in neuroscience journals based on such procedures might be false positives, which would translate into increased proportion of incorrect claims in the literature. However, recent studies have shown that such a problem may originate from the misuse of SPM’s default setting for normalization, 2 × 2 × 2 mm^3^ for a voxel size, instead of RFT itself (Flandin and Friston, 2017; Mueller et al., 2017). They suggested SPM users use an alternative setting for normalization, 3 × 3 × 3 mm^2^, instead of the default setting. Still, the proposed method and recommendation are also based on *P*-values, sharing the same fundamental shortcomings of *P*-values, which are difficult to address by simply changing the way thresholding is done.

To date, most proposed methods have tried to address those problems by changing the way how thresholding is done based on *P*-values. However, to achieve a more fundamental improvement, we propose and advocate a more radical change: abandon *P*-values and switch to a completely different statistical framework: Bayesian hypothesis testing. Problems associated with the use of *P*-values abound (Cohen, 1994; Gigerenzer, 2004; Wagenmakers, 2007; Head et al., 2015; Trafimow and Marks, 2015; Wasserstein and Lazar, 2016); we will not discuss them in detail here, except for one point, the potential benefit of adopting the Bayesian approach in addressing the aforementioned issues of multiple comparison correction.

One might be interested in seeing how Bayesians deal with the problems of type I/II errors. Regarding this, we would like to point out that such errors-oriented framework basically comes from the frequentist school, not the Bayesian one (Gelman et al., 2012). The difference between the two comes from the different ways how they see parameters. Frequentists assume that parameters are ‘fixed but unknown constants.’ Indeed, to define type I and II errors, one has to fix the effect size beforehand. Effect sizes are not random; they must be assumed to be either zero or not (Ellison, 1996). Depending on the assumption, one can define and calculate type I or II error rates. However, this is not the case in Bayesian statistics. For a Bayesian, everything is basically random. Parameter values, or effect sizes, cannot be assumed to be exactly equal to some value (Ellison, 1996). The effect size of interest is not either zero or non-zero for sure. From the very beginning, therefore, one can see that type I/II error framework, which assumes that the parameter is exactly equal to some value, is meaningless in the Bayesian framework (Gelman et al., 2012). Instead, Bayesians express the uncertainty about the effect size in the form of probability distribution. (Woolrich, 2012). Before observing the data, it is called a ‘prior distribution.’ It is ‘updated’ after observing the data; the resulting, updated distribution is called a ‘posterior distribution.’ Still, there is uncertainty about which hypothesis, the null or the alternative, is true (Rouder et al., 2009).

Because this alternative framework does not need to make strong assumptions about effects that are certainly zero for null hypotheses, it is perhaps relatively free from the issue of inflated false positives pertaining to inflated rates of type I errors, and might not strongly require multiple comparison corrections during statistical tests as the frequentist framework does (Gelman et al., 2012). This is one of the reasons why we advocate the use of Bayesian inference in fMRI analysis.

The workhorse for hypothesis testing in the Bayesian framework is called ‘Bayes Factor’ (BF). BF is a measure of statistical evidence in Bayesian statistics, an alternative statistical framework to the currently mainstream frequentist school. BF is currently implemented in SPM version 12. Researchers can easily compute it without much statistical and computational knowledge. BF has several advantages when compared to *P*-values, which we will discuss in the next section, particularly in the cases of studies with small, underpowered samples, which are prevalent in social and cognitive neuroscientific studies (Han and Glenn, 2017).

### Bayes Factors

At the heart of any Bayesian analysis is Bayes’ theorem, which is formulated as follows:

$$\begin{array}{c} P(H\text{V}D)=\frac{P(D|H)}{P(D)}\times P(H) \end{array}$$

where *H* denotes a hypothesis and *D* the data. The term at the left-hand side is called ‘posterior probability,’ which represents the updated belief in *H* after observing *D*. The expression on the right-hand side reveals that the posterior probability is a function of the following terms: *P*(*H*), *P*(*D*|*H*), and *P*(*D*). *P*(*H*) is called ‘prior probability,’ which denotes the belief in *H* before observing the data. And *P*(*D*|*H*) is called ‘likelihood,’ which is the probability of observing the data given that *H* is true. Lastly, *P*(*D*) is called ‘marginal probability,’ which is simply the normalizing constant of the numerator. In a nutshell, Bayesian inference can be seen as the process where the initial belief about *H, P*(*H*), is ‘updated’ to be the new belief in *H, P*(*H*|*D*), by means of applying Bayes’ theorem, hence the term ‘Bayesian updating.’

If we apply Bayes’ theorem in the case where two mutually exclusive and collectively exhaustive hypotheses, say *H*_0_ and *H*_1_, are present, we can obtain the ratio of the posterior probabilities of *H*_0_ and *H*_1_, namely, the posterior odds, which are expressed as follows:

$$\begin{array}{c} \frac{P(H_{1}\text{V }D)}{P(H_{0}\text{V }D)}=\frac{P(H_{1})}{P(H_{0})}\times \frac{P(D\text{V}H_{1})}{P(D\text{V}H_{0})} \end{array}$$

where the marginal probabilities of the numerator and the denominator, *P*(*D*), cancel each other. The second term of the right-hand side, BF_10_= *P*(*D*|*H*_1_)/*P*(*D*|*H*_0_), is called the *Bayes Factor* (Kass and Raftery, 1995). A BF can be conceived as the ratio of the amount of evidence the data provides for H_1_ and H_0,_ or vice versa, depending on the definition of the BF. (Note that we could have defined the Bayes Factor in another way: BF_01_= *P*(*D*|*H*_0_)/*P*(*D*|*H*_1_).) One can verify that the posterior odds, *P*(*H*_1_|*D*)/*P*(*H*_0_|*D*), are obtained by multiplying BF to the prior odds, *P*(*H*_1_)/*P*(*H*_0_). Thus, a BF is a multiplicative factor that is used to update the prior odds to be the posterior odds. Some authors have provided guidelines for interpreting the values of Bayes Factors (Jeffreys, 1961; Kass and Raftery, 1995).

In fMRI analysis, a null hypothesis, H_0_, can be defined in terms of whether significant activity exists in a voxel in the case of one-sample *t*-tests that were performed in the present study. More specifically, because we were mainly interested in comparing activity between two different conditions, we set H_0_ in terms of whether there was a significant difference in activity in the voxel between the two conditions. Hence, H_0_ and H_1_ can be defined as follows:

H_0_: Activity in the voxel in condition A IS NOT greater (or smaller) compared with that in condition B.
H_1_: Activity in the voxel in condition A IS greater (or smaller) compared with that in condition B.

Thus, Bayesian inference in fMRI analysis is to test whether and how strongly the observed functional neuroimaging data supports H_1_ instead of H_0_ in the voxel.

We advocate the use of Bayes Factors over *P*-values for hypothesis testing in fMRI studies on several grounds. First, interpretations of BFs or posterior probabilities are clearer and more straightforward than that of *P*-values, which have been notorious for their difficult interpretation. The misconception that they represent the posterior probability of *H*_0_, *P*(*H*_0_|*D*), is so entrenched among researchers that it seems very difficult to remove (Gigerenzer, 2004; Nuzzo, 2014). But even the correct interpretation is elusive; a *P*-value, the probability that one will observe values of the test statistic that are as extreme or more extreme than is actually observed does not directly quantify the likelihood that *H*_0_ or *H*_1_ is correct. But this is exactly what posterior probability is about. Second, BFs allow us to accept a hypothesis, contrary to the case of *P*-values. Indeed, introductory statistics textbooks teach us that, in principle, we cannot *accept* the null or the alternative; we only reject the null or not. As researchers, however, we are sometimes interested in literally accepting the null or the alternative, so such limited legitimate uses of *P*-values are unsatisfactory. In contrast, Bayes Factors can be used to accept a hypothesis (Rouder et al., 2009). They denote the ratio of the posterior probability of H_1_ to that of H_0_, or vice versa, which can be readily interpreted as ratio of the probabilities that H_0_/H_1_ is true, which can be used to make a decision on whether or not to accept the null of the alternative. This is a more satisfactory way of reaching scientific conclusions than using *P*-values. Fourth, by choosing appropriate priors, it is possible to avoid inadvertently capitalizing on chance. The most common form of this is known as *P*-hacking, where researchers engage in various questionable research practices only to obtain small *P*-values. It will be shown later that such an attempt is not likely to succeed when BFs are used.

### The Current Study

We aim to examine whether Bayesian inference implemented in fMRI analysis software, SPM 12 in particular, produces better results compared with classical frequentist inference. We are particularly interested in the utilization of BFs as indicators in second-level analysis examining activity in brain regions. Although FMRIB’s Software Library (FSL) also implements Bayesian inference based on Markov Chain Monte Carlo (MCMC) sampling, we could not verify that FSL uses BFs for thresholding in second-level inferences (Woolrich, 2012; Webster, 2017). Hence, we focused on SPM 12, which is equipped with thresholding with Bayes Factors in the present study.

Although Bayesian inference in second-level fMRI analysis has the aforementioned benefits compared with frequentist inference, there have been few previous studies addressing topics related to Bayesian inference in SPM. Of course, Bayesian statistics have been widely utilized for parameter estimation and model selection in dynamic causal modeling (Friston et al., 2003). Some previous studies have discussed the statistical foundations of Bayesian inference for both first- and second-level analysis (Friston and Penny, 2003; Neumann and Lohmann, 2003; Penny and Flandin, 2005; Penny and Friston, 2007; Magerkurth et al., 2015; Sidén et al., 2017) and practical guidelines for first-level analysis in SPM (Friston and Penny, 2003; Penny, 2005; Penny and Flandin, 2005; Penny and Friston, 2007). However, none of these studies have suggested practical guidelines for using Bayesian inference for second-level analysis in SPM 12, such as the determination of threshold values that might be informative to end users who do not have profound knowledge in statistics, let alone Bayesian statistics.

Hence, we aim to provide practical guidelines for Bayesian second-level analysis in SPM 12 with comparisons between results from Bayesian and classical inference. Instead of delving into statistical details, we intend to examine Bayesian inference implemented in SPM 12 from the perspectives of end users in order to provide them and potential readers of the present study with insights into how to utilize Bayesian methods in their research. Thus, we follow guidelines suggested by the official manual (Ashburner et al., 2016) and use the default values set by the software whenever possible, because end users are most likely to stick to them in their analyses (Woo et al., 2014). Of course, recent studies have shown that SPM 12’s default settings, a voxel size for normalization, may significantly contribute to the inflation of false positives and other issues, and argued that such settings should not be used automatically (Flandin and Friston, 2017; Mueller et al., 2017). However, in the present study, we aimed to start with default settings, because the majority of SPM users are likely to utilize default settings for their research as Woo et al.’s (2014) survey showed. In addition, we decided to use such default settings for fair comparisons between inference methods with widely used parameters. By starting with default settings, we will be able to provide practical insights and guidelines that can be conveniently implemented to end users of SPM 12.

Because our ultimate aim in the present study is to show how to perform Bayesian inference with SPM 12 to end users, we intended to provide them with concrete examples, actual Bayesian analyses with fMRI data available to public. To this end, we first reanalyzed fMRI data collected for a previous moral psychology research project using the Bayesian second-level analysis procedure implemented in SPM 12 (Han et al., 2016; Han and Glenn, 2017). While explaining our methodology in this article, we showed screenshots from SPM 12 with details directions to provide end users with tutorials to practice Bayesian inference with public fMRI data. Second, we compare results from Bayesian second-level analysis and those from classical second-level analysis by examining survived voxels, as is done in previous studies (Eklund et al., 2016; Han and Glenn, 2017). Third, we examine whether BFs can be better indicators for thresholding compared with *P*-values or *t*-values used in frequentist inference by comparing those indicators by varying the number of subjects whose data are entered into the analyses. The comparisons and tests were conducted to show potential outcomes of Bayesian inference to end users with concrete examples. Finally, we discuss the practical implications of Bayesian inference in second-level fMRI analysis from the perspective of end users. We also suggest some practical guidelines for Bayesian second-level analysis and discuss how the current fMRI analysis tools should be updated to implement Bayesian inference more properly.

## Methods

### Subjects and Materials

We reanalyzed a previously collected moral psychology fMRI dataset with classical and Bayesian inference in the present study (Han et al., 2016; Han and Glenn, 2017). The original data was collected and reanalyzed based on protocols approved by Stanford University IRB (Protocol ID: 25544) and the University of Alabama IRB (Protocol ID: EX-16-CM-083). The fMRI data were initially acquired from 16 participants (8 females) from Northern California. They ranged in age from 21 to 34 years (*M* = 28.59, *SD* = 3.18). They were asked to solve a set of moral and non-moral dilemmas consisting of 60 dilemmatic stories that had been previously invented for fMRI experiments (Greene et al., 2001, 2004). The dilemma set consisted of three different types of dilemmas: 22 moral-personal (MP), 18 moral-impersonal (MI), and 20 non-moral (NM). The MP dilemmas were designed to induce negative intuitive emotional responses in participants by presenting salient potential harm to human beings. The MI dilemmas also required participants to make moral decisions but were designed not to induce immediate gut-level reactions. The NM dilemmas included simple mathematical problems that did not involve any moral judgment. Participants were asked to make a decision about whether a presented behavioral solution was appropriate after reading each dilemma story. Functional images were scanned using a spiral in-and-out sequence with TR = 2000 ms, TE = 30 ms, and flip angle = 90° (Glover and Law, 2001). For each functional scan, a total of 31 oblique axial slices were scanned parallel to the anterior commissure–posterior commissure line with a 4-mm slice thickness and a 1-mm inter-slice skip. The image resolution was 3.75 × 3.75 mm^2^ (field of view = 240 × 240 mm^2^, 64 × 64 matrix).

In addition to the reanalysis of moral psychology fMRI data, we replicated the analysis with three additional datasets containing data collected from 16 or more participants, available at NeuroVault^1^ (Gorgolewski et al., 2015), which is an open repository for image files containing results from statistical analyses. We downloaded image files the from three data collections containing results from first-level analyses examining the neural correlates of various cognitive processes (e.g., mental calculation, face recognition) at the within-subject level (Henson et al., 2002; Amalric and Dehaene, 2016; Gordon et al., 2017; Kievit et al., unpublished). Further details, including the NeuroVault ID, citation information, analyzed contrast, number of included images, and type of statistical map for each dataset are presented in **Table 1**.

**Table 1:** Previous studies analyzed for replications.

NeuroVault ID	Study	Task	Images (*N*)	Type	Clusterwise	Voxelwise	Bayesian
1160	Kievit et al., unpublished	Raven’s Matrices difficulty (correlation)	35	*T*-map	968	30	210
1805	Amalric and Dehaene, 2016	Equation versus baseline	29	*Z*-map	14,173	4,987	9,064
1811	Henson et al., 2002	Famous face versus baseline	16	*Z*-map	6,507	1,085	4,441
2447	Gordon et al., 2017	Face versus word	10	*T*-map	992	2	33

### Procedures

#### Preprocessing and First-Level Analysis

The scanned images were analyzed using SPM 12. First, we performed RETROICOR (Retrospective Image Correction) and RVHRCOR (respiration variations and heart rate correction) to minimize artifacts associated with respiratory and cardiac activities (Glover et al., 2000; Chang and Glover, 2009). These corrections were performed by using a LINUX script provided by Glover (2009), while spiral in-and-out functional images were being reconstructed. Second, we conducted slice time correction, motion correction, co-registration with structural images, normalization into SPM’s standard MNI space (79 × 95 × 68, 2 × 2 × 2 mm^3^ voxels), and spatial smoothing with Gaussian FWHM = 8 mm. All these preprocessing procedures were performed following steps and using parameters suggested in tutorials in the SPM 12 manual (Ashburner et al., 2016) and with SPM’s default settings.

We conducted first-level analysis for each participant with the preprocessed images. For the first-level analysis, the regressors for the corresponding dilemma type blocks were modeled as a boxcar function convolved with the canonical hemodynamic response function. For each trial, we modeled neural activity, four scans before, one during, and three after the moment of response, similar to the analyses conducted by Greene et al. (2001, 2004). We treated each voxel according to SPM’s general linear model. The first-level analysis was also performed following the guidelines and parameters provided by SPM tutorials and with SPM’s default values (Ashburner et al., 2016). Once the first-level analysis was completed, we created a contract image, moral (MP + MI) versus NM conditions, for each participant for the second-level analysis. In addition to the main contrast of interest, moral versus non-moral conditions, we created four different types of contrasts, MP versus NM, MI versus NM, MP versus MI and MI versus MP conditions, for exploratory purposes.

#### Second-Level Analysis

We performed the second-level analysis with classical and Bayesian inference implemented in SPM 12. To examine the difference in neural activity between moral and NM conditions, we performed a one-sample *t*-test with contrast images created from first-level analysis. All 16 contrast images were entered into a second-level one-sample *t*-test model. Then, we first used a classical inference module implemented in SPM 12. At the end of the classical inference, we examined which voxels survived with thresholds provided by SPM 12 by default. The following thresholding criteria were utilized: (1) a cluster-forming threshold *p* < 0.001 and a cluster-wise threshold *p* < 0.05 [familywise error (FWE) corrected] and (2) a voxel-wise threshold *p* < 0.05 (FWE corrected).

**FIGURE 1:**
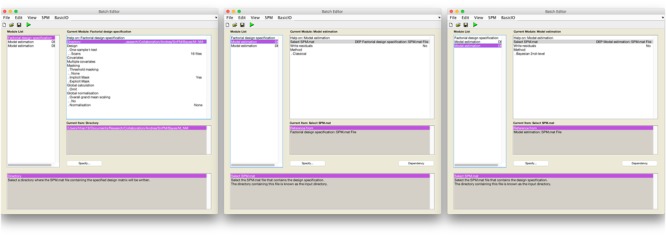
Batch editor settings for Bayesian second-level inference in SPM 12.

Secondly, we used a Bayesian second-level inference module by setting a dependency on the output from the classical inference module. The processed SPM.mat from the classical inference model was used for the input for Bayesian second-level inference (see **Figure 1** for screenshots from the batch editor with Bayesian inference modules). End users may follow these steps to conduct the same second-level inference with the fMRI data downloaded from NeuroVault with SPM 12. First, a “factorial design specification” module should be added to SPM 12’s batch editor. For the design, “one-sample *t*-test” should be enabled. Similar to usual one-sample *t*-test cases, users may simply choose contrast images produced from the prior first-level analysis, sixteen moral psychology fMRI image files shared via NeuroVault in the case of the present study. Once the design is specified, a “model estimation” module should be added. Similar to usual classical inference cases, users should set “SPM.mat” as “dependency: DEP Factorial design specification,” and select “classical” for the method. Before performing Bayesian inference, classical inference should be completed to calculated values required for the Bayesian inference. Finally, users should add one additional “model estimation” module in the batch editor. In this second “model estimation” module, the input “SPM.mat” should be “dependency: DEP model estimation,” and “method” should be “Bayesian 2nd-level.” Once this batch job is completed, SPM 12 will create a SPM.mat file containing outputs both from classical and Bayesian second-level inference.

**FIGURE 2:**
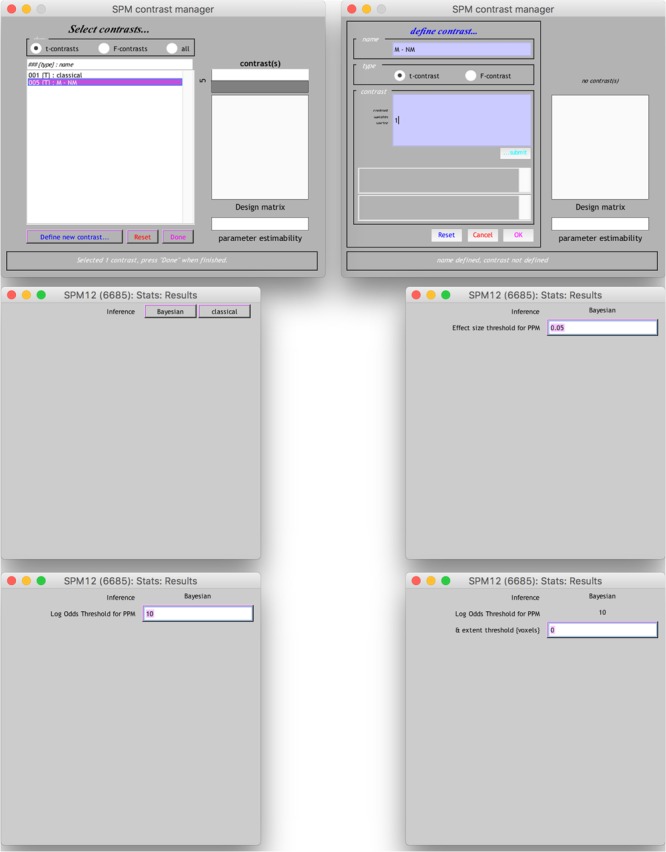
Contrast manager settings for Bayesian second-level inference in SPM 12.

**FIGURE 3:**
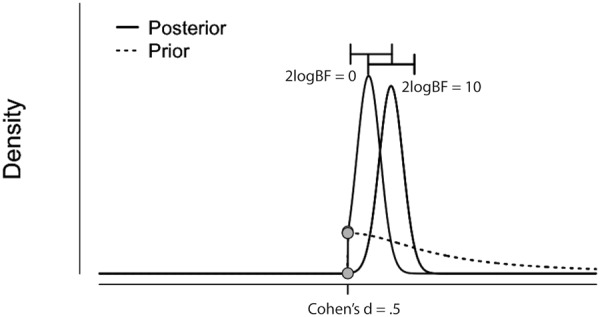
Illustrative example showing prior (dashed curve) and posterior distributions when 2logBF = 0 (left filled curve) and 2logBF = 10 (right filled curve), and ES threshold is Cohen’s *d* = 0.5. Figure originally created by JASP and visualized by Adobe Illustrator. H0: a difference in activity between conditions A and B IS NOT GREATER than *d* = 0.5. H1: a difference in activity between conditions A and B IS GREATER than *d* = 0.5.

The results from the Bayesian second-level inference were thresholded with the following criteria: (1) an effect size (ES) threshold and (2) a logBF (natural logarithm of Bayes Factor) threshold (see **Figure 2** for screenshots from the results window demonstrating how to set thresholds). The default EF threshold value was a square root of the prior parameter covariance stored in SPM.PPM.cB, a variable storing the square of the conditional standard deviation of the prior parameter. In terms of ES, the square root of SPM.PPM.Cb can be understood as Cohen’s *d* = 1.0. In the base of the BF threshold, the default value is logBF > 1.0. In addition to these SPM 12 default thresholds, we used other threshold values based on prior statistical knowledge. For ES thresholds, we used *d* > 0.2 (0.2$\begin{array}{c} \sqrt{SPM.PPM.cB} \end{array}$), >0.5 (0.5$\begin{array}{c} \sqrt{SPM.PPM.cB} \end{array}$) and >0.8 (0.8$\begin{array}{c} \sqrt{SPM.PPM.cB} \end{array}$), corresponding to a small, medium, and large ES, respectively (Cohen, 1992). For the logBF thresholds, we employed logBF > 3 and > 5, corresponding to the presence of evidence strongly supporting H_1_: voxel contrast > ES threshold, instead of H_0_: voxel contrast ≤ ES threshold, very strongly supporting H_1_ instead of H_0_. An illustrative example is presented in **Figure 3**. **Figure 3** shows the prior and posterior distributions when *d* = 0.5 is set as an ES threshold. Posterior distributions plotted in dashed lines show how posteriors would be distributed when 2logBF = 0 (no evidence supporting H_1_) and 2logBF = 10 (logBF = 10, very strong evidence supporting H_1_). Although SPM 12 asks a voxel-extent threshold for Bayesian inference similar to classical inference, we did not set any specific voxel extent threshold in the present study, because there was not any algorithm for the calculation of clusterwise threshold, such as FWE and FDR in classical inference, implemented in Bayesian inference. Thus, we set the voxel extent threshold as 0 similar to the case of usual voxelwise classical inference.

End users may also conduct thresholding with a “SPM contrast manager” module in SPM 12, which can be opened by clicking “Results” button on SPM 12’s main menu. Once a contrast manager appears, readers should create a new *t*-contrast with a contrast value of “1” to examine which voxels show positive activity. When readers are asked to choose an inference method, they should choose “Bayesian” to enter thresholding parameters for Bayesian inference. First, the contrast manager requires the ES threshold value. In the text box, an ES value equivalent to Cohen’s *d* = 1.0 (e.g., 0.0518 in the case of moral psychology fMRI data^2^) appears as a default value. Readers may enter an ES value (e.g., 0.0259 in the case of moral psychology fMRI data for Cohen’s *d* = 0.5^3^) following the guidelines related to Cohen’s *d*. Then, the second thresholding parameter, “Log odd Threshold,” logBF, should be entered. Although SPM 12 suggests 10 as a default value, readers may use 3 (presence of strong evidence supporting H_1_) or 5 (presence of very strong evidence H_1_). Finally, “extent threshold (voxel)” may be left 0, because a voxel-extent threshold will not be considered in our guidelines as mentioned previously. Once all thresholding parameters are entered, readers will be able to see a result screen showing survived voxels and related information, such as voxel coordinates and cluster sizes.

In addition to the second-level analyses of the contrast of moral versus non-moral conditions, we conducted the same analyses for four other contrasts, MP versus NM, MI versus NM, MP versus MI, and MI versus MP conditions. For the aforementioned contrasts, we performed both classical and Bayesian second-level analyses with the same threshold used for the analyses of the moral versus NM contrast.

#### Result Comparison

We compared outcomes from Bayesian second-level analysis with those from classical inference with SPM’s default methods for multiple comparisons and clusterwise and voxelwise FWE. As we mentioned in the introduction, the most widely used default setting, clusterwise FWE implemented in SPM, was found to produce inflated false positives (Eklund et al., 2016). On the other hand, voxelwise FWE was reported to control for false positives well; however, this correction method occasionally adjusted the familywise error rate, which was supposed to be 5%, to lower than 5% and produced conservative outcomes (Eklund et al., 2016). Thus, we examined how the outcomes from Bayesian second-level analysis were different from those from classical inference with the aforementioned correction methods supported by SPM 12.

First, we counted the number of survived voxels as an indicator for whether a specific method produced was sensitive or conservative, as Han and Glenn (2017) did. By using a customized MATLAB code, we calculated the number of voxels that exceeded the *t*-value threshold (for classical inference) or ES/logBF thresholds (for Bayesian inference). Because there is no feasible way to set “true activations” and test sensitivity and selectivity based on such true activations while evaluating Bayesian inference, which is based on different statistical foundations and assumptions compared with classical inference, we decided to use the number of survived voxels to approximately evaluate the sensitivity and selectivity of Bayesian inference. Because Eklund et al. (2016) reported that classical inference with clusterwise FWE may produce more survived voxels than expected and that with voxelwise FWE may be overly conservative, we examined whether the number of survived voxels resulting from Bayesian inference was situated somewhere between those two extreme cases and which of the ES and logBF thresholds produced such a result. We assumed that Bayesian inference can produce better outcomes compared with the aforementioned two other inference methods if the resultant number of survived voxels from Bayesian inference is smaller than that from clusterwise FWE inference and is greater than that from voxelwise FWE inference. We investigated the numbers of survived voxels for all five analyzed contrasts.

Second, in addition to the aforementioned comparisons of the numbers of survived voxels, we examined whether Bayesian inference was robust against possible noises for an exploratory purpose. Because assuming true signals and examining FDRs or something similar might not be an optimal way to evaluate the performance of Bayesian inference given the philosophical aspects of its methodology, such as the definition of Bayes factors (Kass and Raftery, 1995; Wagenmakers et al., 2017), we decided to focus on whether Bayesian inference can produce consistent outcomes while being influenced by possible random noises. First, to create images containing random noises, we added a Gaussian noise (*SD* = approximately 25% of the mean signal strength in each first-order image) to each first-order image; we decided to adopt the 0.25 *SD* as a parameter in this process following the random noise parameter used for the second-level analyses in Woo et al. (2014). We repeated this process ten times. Second, we conducted the same Bayesian and classical inferences with the images containing random noises. Third, we calculated two values, *false alarm* and *hit* rates, to compare outcomes between Bayesian and classical inferences. The *false alarm* rate is defined as the ratio of voxels marked as active from the analysis of noise-added images but as inactive from the analysis of original images to voxels marked as active from the analysis of noise-added images. The *hit* rate is defined as the ratio of voxels marked as active from both analyses to voxels marked as active from the analysis of the original images. These *false alarm* and *hit* rates indicators seem similar to a FDR and sensitivity, respectively (Nichols and Hayasaka, 2003; Woo et al., 2014), in terms of methods of calculation. However, because we did not assume true signals or activations in the present study, *false alarm* and *hit* rates are not identical to a false discover rate and sensitivity, which are based on frequentist assumptions. We assumed that the lower *false alarm* rate and higher *hit* rate indicate a better performance. Comparisons between inference methods were performed with Bayesian ANCOVA implemented in JASP (Love et al., 2017). We used 2logBF ≥ 2 (Kass and Raftery, 1995), indicating the presence of positive evidence supporting H_1_ instead of H_0_, as a threshold for the presence of actual effects or differences in Bayesian ANCOVA.

Third, we tested whether entering different numbers of contrast images from the first-level analysis into the second-level analysis model produced different outcomes in terms of *t* (for classical inference) or logBF values (for Bayesian inference) for the two inference methods. We were particularly interested in whether Bayesian inference was more robust against the input of subject data that might contain outliers compared with classical inference. Thus, we counted the number of survived voxels resulting from different inference methods while the sample size was increasing. We first entered two randomly selected contrast images to the second-level analysis models. Then, additional contrast images were entered into the models. We examined the number of survived voxel numbers for each inference method every time an additional contrast image was entered in the second-level analysis. This process was repeated ten times to acquire data with different randomized orders of contrast image entering. The order of first-level contrast images entered into the model for each trial is presented in Supplementary Table S1.

Furthermore, we compared the statistical scores at (-4, 48, 12) calculated by the aforementioned two inference methods. This voxel was selected because first, it survived both analyses with SPM 12’s default thresholding settings; second, the previous meta-analyses of fMRI studies of moral psychological experiments reported a common activation in the voxel (Eres et al., 2017; Han, 2017). Following the same randomization procedure explained previously, the *t*-value and logBF value in the aforementioned voxel were calculated when each additional image was entered. Changes in these values across the different sample sizes were plotted for comparisons between inference methods. Moreover, we performed Bayesian repeated measures ANOVA implemented in JASP (Love et al., 2017) to examine the pattern of the changes. Finally, in order to test whether the statistical indicator type (*t* versus logBF) influenced the relationship between the statistical indicator values and the sample size, we performed two-level Bayesian repeated measures ANOVA. We conducted this test only for the contrast of moral versus NM conditions, which was the main contrast of the present study.

#### Replication

To examine whether Bayesian inference can be applicable to fMRI datasets other than the aforementioned moral psychology fMRI dataset, we replicated our analyses with three additional datasets downloaded from NeuroVault. Because these datasets contained results from the first-level analyses, we conducted classical and Bayesian second-level analyses with downloaded image files. Following the same procedures described above, we calculated the number of voxels that survived classical clusterwise inference, classical voxelwise inference (with FWE correction), and Bayesian inference. We compared these numbers across different inference methods in order to examine their selectivity and sensitivity. We also compared the *false alarm* and *hit* rates among the different inference methods.

## Results

### Number of Survived Voxels

**FIGURE 4:**
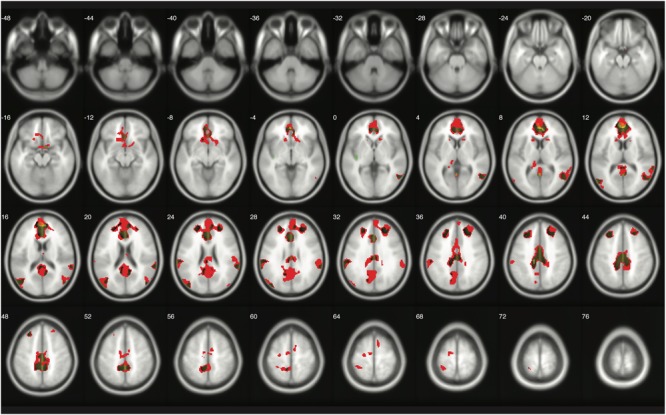
Results from classical and Bayesian second-level inferences for the contrast of moral versus non-moral task conditions. Images created by XjView (Cui et al., 2015). Red: classical clusterwise inference. Yellow: classical voxelwise FWE inference. Green: Bayesian inference.

The number of survived voxels resulting from the classical and Bayesian second-level analyses are presented in **Table 2**. Among all the cases with different ES and logBF thresholding settings, the number of survived voxels resulting from Bayesian inference was smaller than that resulting from clusterwise FWE inference, and was larger than that resulting from voxelwise FWE inference when *D* = 0.2 and logBF = 5, *D* = 0.5 and logBF = 5, *D* = 0.8 and logBF = 3, *D* = 0.8 and logBF = 10, and *D* = 1.0 and logBF = 0.3 in all five contrasts. Given these, when moderate thresholds (a medium ES, *D* = 0.5, and logBF = 5, indicating the presence of very strong evidence supporting H_1_ instead of H_0_) were applied, Bayesian second-level analysis was more conservative than clusterwise FWE inference while being more sensitive than voxelwise FWE inference (see **Figure 4** for analysis results with the contrast of moral versus NM conditions with the aforementioned criteria). As the *D* and logBF threshold increased, the number of survived voxels decreased.

**Table 2:** Number of survived voxels across different contrasts, inference methods, and parameters.

	Classical		Bayesian											
	Clusterwise	Voxelwise	*D* = 0.2, logBF = 3	*D* = 0.2, logBF = 5	*D* = 0.2, logBF = 10	*D* = 0.5, logBF = 3	*D* = 0.5, logBF = 5	*D* = 0.5, logBF = 10	*D* = 0.8, logBF = 3	*D* = 0.8, logBF = 5	*D* = 0.8, logBF = 10	*D* = 1, logBF = 3	*D* = 1, logBF = 5	*D* = 1, logBF = 10
MP + MI - NM	14,424	264	24,557	9,645	1,107	13,492	4,952	309	7,038	2,100	45	4,892	1,236	17
MP – NM	13,540	817	25,623	13,055	3,245	14,751	7,007	1,589	8,144	3,511	666	5,885	2,195	386
MI – NM	7,458	0	6,056	367	0	2,128	64	0	412	2	0	144	0	0
MP – MI	2,941	46	5,237	1,365	99	2,461	601	26	1,097	242	2	536	120	0
MI – MP	6,826	22	11,959	2,855	68	5,388	1,004	15	2,204	265	2	857	95	0

### False Alarm and Hit Rates

**FIGURE 5:**
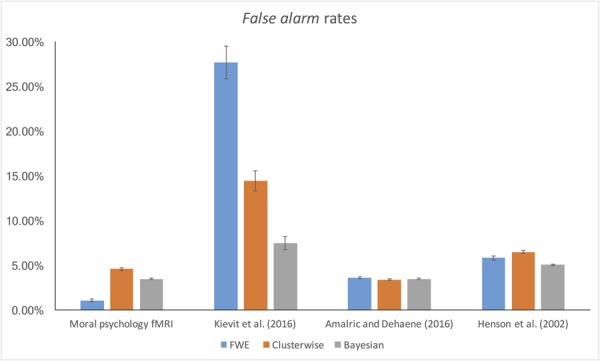
*False alarm rate* calculated from moral psychology fMRI data.

**FIGURE 6:**
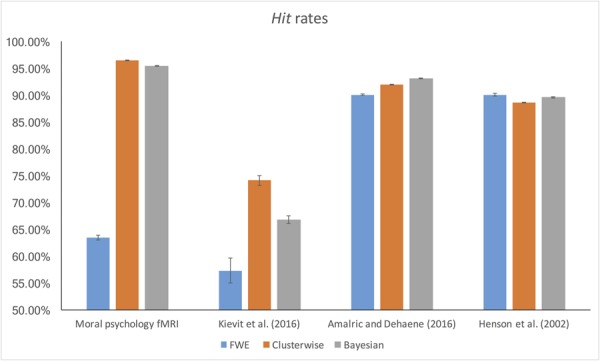
*Hit rate* calculated from moral psychology fMRI data.

The *false alarm* and *hit* rates calculated from moral psychology fMRI data are demonstrated in **Figures 5, 6**, respectively. Bayesian ANCOVA indicated that the effect of inference type was significant in the analyses of both the *false alarm* (2logBF = 58.35) and *hit* (2logBF = 130.31) rates. In the case of the *false alarm* rate, *post hoc* comparisons demonstrated that voxelwise FWE inference outperformed both Bayesian (2logBF = 32.32) and clusterwise (2logBF = 39.97) inferences. Bayesian inference showed a better performance compared with clusterwise inference (2logBF = 16.90). In the case of the *hit* rate, clusterwise inference showed a better performance compared with both Bayesian (2logBF = 22.16) and voxelwise FWE (2logBF = 81.80) inference. Bayesian inference outperformed voxelwise FWE inference (2logBF = 80.56). These results show that Bayesian inference performed better than clusterwise inference in terms of the *false alarm* rate, and voxelwise FWE inference performed better in terms of the *hit* rate.

### Changes in Analysis Results across Different Sample Sizes

#### Changes in Number of Survived Voxels

**FIGURE 7:**
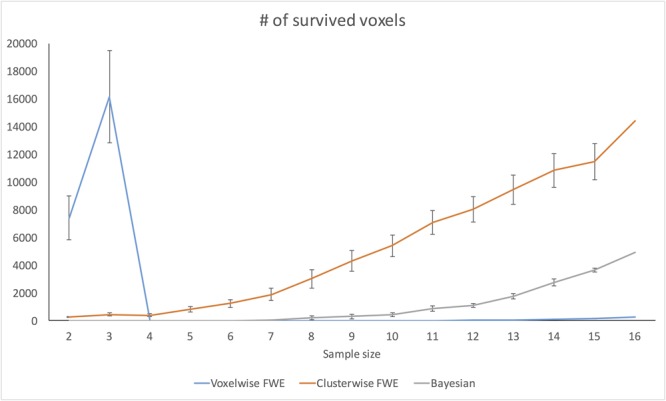
Changes in survived voxel numbers with different inference methods across different sample sizes.

**FIGURE 8:**
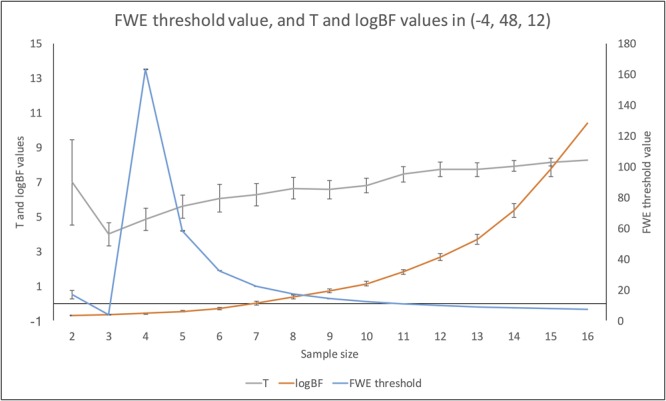
Changes in FWE threshold value, and *t-* and logBF values in (–4, 48, 12) across different sample sizes.

**Figure 7** demonstrates changes in the number of survived voxels resulting from Bayesian second-level analysis and clusterwise and voxelwise FWE inference. The numbers from Bayesian and clusterwise FWE inference showed monotonically increasing patterns. Interestingly, although the number from voxelwise FWE showed an immediate increase when the sample size increased from two to three, it decreased steeply when the sample size again increased to four. It started to increase monotonically thereafter. This trend in the case of voxelwise FWE might be associated with the change in the voxelwise FWE-corrected threshold. As shown in **Figure 8**, this threshold value showed a radical change when the sample size increased from two to four. When the sample size was greater than five, the threshold value showed a steady decline that was consistent with the aforementioned steady increase in the number of survived voxels.

#### Changes in *t*- and logBF Values in (-4, 48, 12)

In addition, we examined how the *t*- and logBF values in (-4, 48, 12) changed when different numbers of contrast images were entered into the analyses. As shown in **Figure 7**, the logBF value showed a steady increase as the sample size increased. We performed Bayesian repeated measures ANOVA with JASP in order to examine whether the patterns of changes in the *t*- and logBF values were significantly different from each other. The result from Bayesian repeated measures ANOVA indicated that the data very strongly supported the main effect of sample size, logBF = 47.76. However, the change in the *t*-value was less continuous than that of the logBF value. The Bayesian repeated measures ANOVA results corroborated this point; the main effect of sample size was verified, but it was not as strong as in the previous case, logBF = 4.77. When we compared the patterns of changes in the *t*- and logBF values, the resultant logBF value suggested very strong evidence supporting that these patterns were significantly different from each other, logBF = 136.82. Given this, the pattern of the change in *t*-values associated with the increase in the sample size was significantly different from that in logBF, which showed a monotonic increase.

### Replications

**FIGURE 9:**
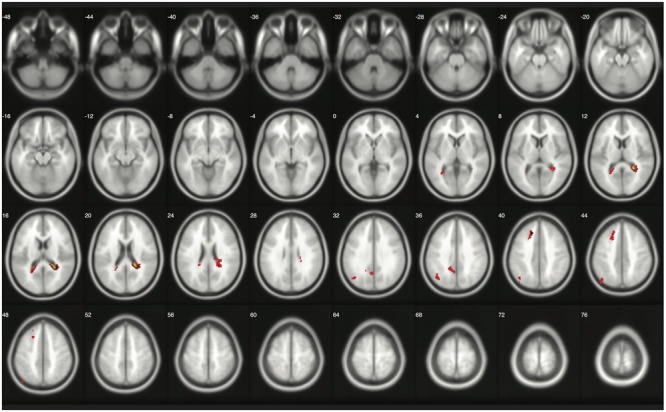
Results from classical and Bayesian second-level inferences with Kievit et al.’s (unpublished) data. Images created by XjView (Cui et al., 2015). Red: classical clusterwise inference. Yellow: classical voxelwise FWE inference. Green: Bayesian inference.

**FIGURE 10:**
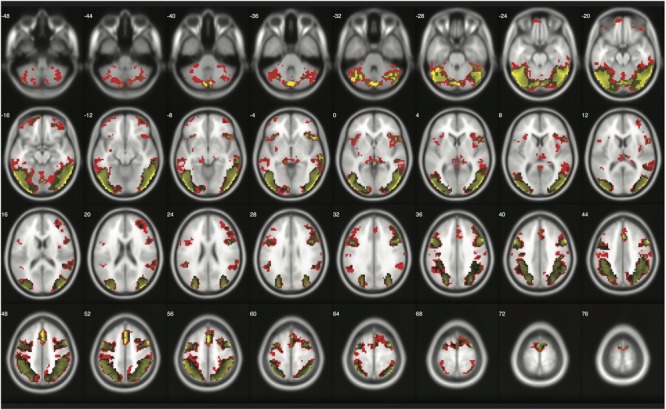
Results from classical and Bayesian second-level inferences with Amalric and Dehaene’s (2016) data. Images created by XjView (Cui et al., 2015). Red: classical clusterwise inference. Yellow: classical voxelwise FWE inference. Green: Bayesian inference.

**FIGURE 11:**
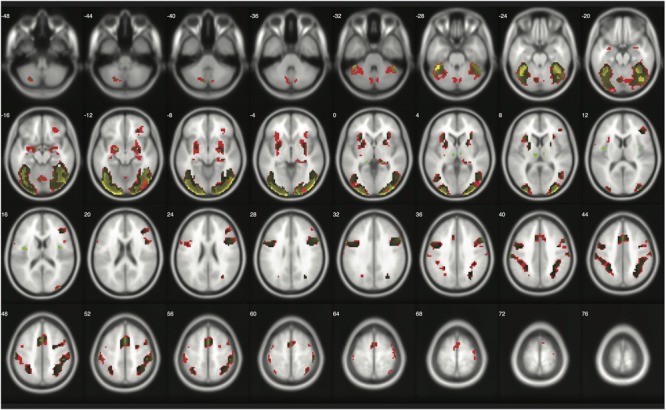
Results from classical and Bayesian second-level inferences with Henson et al.’s (2002) data. Images created by XjView (Cui et al., 2015). Red: classical clusterwise inference. Yellow: classical voxelwise FWE inference. Green: Bayesian inference.

When moderate Bayesian thresholds (a medium ES, *D* = 0.5, and logBF = 5) were applied, Bayesian inference showed better selectivity than classical clusterwise inference with all four datasets analyzed for replications, while showing better sensitivity than classical voxelwise inference; in other words, the number of survived voxels were classical clusterwise inference > Bayesian inference > classical voxelwise inference (see **Table 1**). We were able to successfully replicate the findings from the reanalysis of the moral psychology fMRI dataset with three additional datasets. **Figures 9–11** compare voxels that survived three different inference methods in the reanalysis of Henson et al. (2002), Amalric and Dehaene (2016), and Kievit et al. (unpublished), respectively.

Furthermore, we examined the *false alarm* and *hit* rates with the additional datasets. The overall results are demonstrated in **Figures 4, 5**. First, in the case of the reanalyses of Kievit et al.’s (unpublished) data, the effect of inference type was significant in ANCOVA for both the *false alarm* (2logBF = 37.25) and *hit* (2logBF = 23.51) rates. Bayesian inference outperformed both clusterwise (2logBF = 11.46) and voxelwise FWE (2logBF = 28.20) inferences in terms of the *false alarm* rate, while clusterwise inference showed a better performance compared with voxelwise FWE inference (2logBF = 15.25). In terms of the *hit* rate, clusterwise inference outperformed both Bayesian (2logBF = 15.61) and voxelwise FWE (2logBF = 16.43), while Bayesian inference outperformed voxelwise FWE (2logBF = 6.49). Second, we found a significant effect of inference type from the ANCOVA of the *hit* rate with Amalric and Dehaene’s (2016) data (2logBF = 77.39); however, such an effect was insignificant in the case of the comparison of the *false alarm* rate (2logBF = -0.95). We could not find any significant differences in the *false alarm* rates between Bayesian inference versus voxelwise FWE inference (2logBF = -1.13), Bayesian inference versus clusterwise inference (2logBF = -1.28), and voxelwise FWE inference versus clusterwise inference (2logBF = 0.03). However, in the case of the *hit* rate, Bayesian inference outperformed both clusterwise (2logBF = 33.24) and voxelwise FWE (2logBF = 55.16) inference, while clusterwise inference outperformed voxelwise FWE (2logBF = 38.92). Third, the effect of inference type was significant in the cases of *false alarm* (2logBF = 18.32) and *hit* (2logBF = 15.82) rates from our reanalyses of Henson et al.’s (2002) data. In the case of the *false alarm* rate, both the Bayesian (2logBF = 23.01) and voxelwise FWE (2logBF = 4.51) inferences outperformed clusterwise inference. Bayesian inference also showed a better performance than clusterwise inference (2logBF = 2.13). In the case of the *hit* rate, both the Bayesian (2logBF = 17.88) and voxelwise FWE (2logBF = 12.32) inferences showed significantly better performances compared with clusterwise inference; however, the difference in the rate between Bayesian and voxelwise FWE inferences was insignificant (2logBF = -0.40).

## Discussion

In the present study, we compared outcomes from classical and Bayesian second-level analyses implemented in SPM 12. We found that first, given the number of survived voxels, Bayesian second-level analysis was more sensitive than voxelwise FWE inference while being more conservative than clusterwise FWE inference when both a medium ES threshold (Cohen’s *D* = 0.5) and a Bayes factor threshold indicating the presence of very strong evidence (logBF = 5) were applied. Given the previous studies examining inflated false positives in fMRI analysis (Eklund et al., 2016; Han and Glenn, 2017), this result suggests that Bayesian inference can control false positive rates better than clusterwise FWE inference while maintaining higher sensitivity than voxelwise FWE inference under appropriate choices of thresholding values.

Second, the results from the comparisons of *false alarm* and *hit* rates also support the aforementioned benefit of Bayesian inference. Although voxelwise FWE inference showed the best performance in the comparison of the *false alarm* rate, Bayesian inference outperformed clusterwise inference. Also, in the case of the *hit* rate, although clusterwise inference showed the best performance, the *hit* rate of Bayesian inference was better than that of voxelwise FWE inference and was very high (>95%). In other words, Bayesian inference was less likely to show activations that did not exist in results from analyses of original images (lower *false alarm* rates), or to ignore activations that did exist in results from analyses of original images (higher *hit* rates), compared with other aforementioned inference methods when random noises present in images in general. These suggest that Bayesian inference can better reproduce results from analyses of fMRI images without any noises (original images) even when random noises present. Given these results, Bayesian inference shows consistent and robust performances with images with possible noises, and its performances might be better or at least similar to those of classical inference methods.

Third, the pattern of change in the *t*-statistics found in the present study suggests that Bayesian inference is more robust to variations in sample sizes than classical inference. Interestingly, the *t*-statistics threshold value after applying voxelwise FWE correction fluctuated significantly when the sample size was very small (*n* < 4). Similarly, our investigation of the changes in *t*- and logBF values following the increase in sample size showed that the logBF value increased steadily, while the *t*-value did not, as the sample size increased. Particularly, when the sample size was very small (*n* < 4), the *t*-value showed a slight decrease. Although researchers may not employ such a very small sample size for their fMRI studies due to the issue of statistical power (Lieberman and Cunningham, 2009; Button et al., 2013), these trends concerning the relationship among ordinary and FWE-corrected *t* statistics, logBF, and sample size imply that Bayesian inference is a more robust inferential method when the sample size is extremely small. This result supports the previous argument about the benefits of Bayesian methods in fMRI studies with small, underpowered samples (Poldrack et al., 2017).

Fourth, concerns related to the *de facto p* threshold for publication, *p* < 0.05, encourage us to utilize the Bayesian approach in lieu of the frequentist approach. A recent study demonstrated that findings sufficing the *p* < 0.05 threshold can merely provide anecdotal or weekly positive evidence at best in supporting H_1_. Thus, it is argued that researchers, particularly those in psychological studies, should adopt *p* < 0.005 as a new threshold for claiming ‘significance,’ while referring to results with a *p* < 0.05 but not *p* < 0.005 threshold as ‘suggestive,’ not ‘significant’ (Button et al., 2013). This requirement would make psychological studies utilizing fMRI data analysis more challenging, because it would require studies to increase their sample size by at least 60% (Button et al., 2013). The issue of correction for multiple comparisons that is prevalent in fMRI analysis also makes the situation worse because a corrected threshold will become even higher (Nichols and Holmes, 2002; Eklund et al., 2016; Han and Glenn, 2017). Bayesian inference would be relatively free from these issues, because a logBF value, which is essentially a Bayes factor, deals with the strength of our belief about the presence of evidence supporting H_1_ with available data (Kass and Raftery, 1995). In contrast, because a *P-*value ‘quantifies the unusualness of the data under the null hypothesis leaving open the possibility that the data are even more likely under a well-specified and plausible alternative hypothesis’ (p. 10), classical inference involves multiple tests and is more susceptible to inflated false positives (Wagenmakers et al., 2017). Although some argue that Bayesian inference may also need correction for multiple comparisons (e.g., Scott and Berger, 2010), it may not be as serious an issue as the case of classical inference because the main interest of Bayesian statistics is the strength of belief in the presence of evidence instead of preventing false positives. Hence, given the issues associated with a *P*-value threshold and correction for multiple comparisons, we recommend the use of Bayesian inference in fMRI analysis. Furthermore, our result supports that Bayesian inference can well control for possible false positives when statistically reasonable ES and BF thresholds were employed, although Bayesian inference is not basically concerned about the issue of inflated false positives (Gelman et al., 2012). In fact, the number of survived voxels from Bayesian reanalysis was smaller than that from clusterwise FWE inference, which was deemed to show an inflated Type I error rate (Eklund et al., 2016). This result suggests that Bayesian inference can implicitly address the aforementioned issue among frequentists perhaps by employing priors instead of frequentist assumptions on true zero effects and BF thresholds (Gelman et al., 2012; Wagenmakers et al., 2017), even if Bayesians are not explicitly concerned about and does not directly control for Type I error.

In addition to these points supporting the practical value of Bayesian inference in second-level fMRI data analysis, we consider its philosophical benefits as well. First, as noted earlier, Bayesian analyses are straightforward to understand because they provide researchers with exactly what they wish to know: *P*(*H*|*D*), the probability that a hypothesis, either null or alternative, is true given the data. In contrast, *P*-values, which frequentist analyses provide, are inverse probabilities of posteriors and, thus, are at best indirectly related to what researchers wish to learn.

Second, Bayes Factors, the Bayesian hypothesis testing method we used in the present study, directly quantify how much a statistical hypothesis is more likely to be true than another one. This feature allows researchers to directly compare statistical models, for example, the null and the alternative, in a way that is readily interpretable. A counterpart in the traditional framework, the likelihood ratio test, pursues a similar goal, but it does not directly compare the probabilities of different hypotheses, nor does it demonstrate to what degree a hypothesis is more likely than another. Unfortunately, even its use has been quite limited in practice due to the prevalence of *P*-values that do not take into account the presence of alternative hypotheses.

Third, if one chooses to employ thresholds, their goals are more straightforward in the Bayesian framework than in the frequentist one. The traditional threshold, namely, ‘*p <* 0.05 (or 0.01),’ is meant to prevent the rate of false positives from being greater than the nominal significance level specified. However, it is silent on the scientific problems themselves: how to choose between hypotheses and why *the* specific threshold value, 0.05 or 0.01, should be used. However, in the case of Bayes Factors, researchers can be clearer about these considerations; they can clarify how they compared the hypotheses and can provide grounds for the specific threshold value for accepting or rejecting the null, or the alternative, hypothesis.

Of course, some may argue that BF interpretation guidelines might also be arbitrary similar to the cases of *P*-value thresholds. However, although BFs might not be completely free from the arbitrariness in interpretation, it is obvious that they are better in decision-making compared with *P*-values and thresholds (Stern, 2016). While thresholding *P*-values requires a binary decision-making, such as accepting or rejecting H_0_, BFs allow us to consider the strength of evidence supporting H_1_ in a continuous manner. Although we need to set a certain threshold for BFs, such as 2logBF = 5, because SPM 12 required us to do so, users can examine the strength of hypothesis-supporting evidence existing in voxels by referring to BFs. In addition, guidelines of BF thresholding are firmly based on empirical grounds pertaining to strength of evidence (Kass and Raftery, 1995), unlike *P*-value thresholds that are difficult to clearly interpret their implications (e.g., how should we interpret the meaning of and what should we do with *p* = 0.051 in a voxel?). Thus, Bayesian inference can be a useful alternative inference method for fMRI analysis to address issues associated with how to do thresholding and make judgments based on *P*-values. Even if Bayesian inference may not be able to immediately replace classical inference that is currently used for fMRI analysis, users may consider reporting results from Bayesian inference in addition to those from classical inference in their papers to provide readers with more and better information for judgments.

Furthermore, we showed that the findings from the reanalysis of a moral psychology fMRI dataset were successfully replicated with three additional datasets and that Bayesian inference can be applicable to various domains of fMRI studies. Also, the results from the comparisons of *false alarm* and *hit* rates suggest that Bayesian inference can work consistently and robustly under the influences of possible noises given its better or at least similar performance compared with classical inference methods. This suggests that various types of fMRI data analyses can benefit from applying Bayesian inference as well, similar to the case of the analysis of the socio-moral fMRI data. The findings from the replications will also contribute to the generalizability of the main findings from the present study.

However, there are several limitations that warrant the necessity of future studies. First, we only examined the case of the simplest second-level analysis, a one-group *t*-test, in the present study. Future studies may need to examine more-complex tests, such as a two-group *t*-test and correlation analysis, to see whether Bayesian second-level analysis implemented in SPM 12 can also be applicable to these tests, which involve multiple covariates. Second, because we aimed to compare the results from Bayesian and classical second-level analysis, we did not examine the case of Bayesian first-level analysis in the present study. It would be informative to examine the combination of Bayesian first- and second-level analysis in future studies. Third, although we proposed some practical guidelines for Bayesian second-level analysis, such as the application of Cohen’s *D* > 0.5 and logBF > 5 for thresholding, more statistical evaluations pertaining to whether these guidelines are reliable and valid need to be conducted. We used the number of survived voxels and the change in statistics with different sample sizes for the evaluation of Bayesian inference, which are very simple indicators for evaluation. Future studies could utilize more-sophisticated evaluation methods, such as simulations (Eklund et al., 2016). Fourth, although we assumed that the number of survived voxels can be used to evaluate sensitivity and selectivity, it might not be an ideal indicator for such an evaluation. Because we could not set true activations due to the mechanism of Bayesian inference, which underscores the presence of supporting evidence in data, we could not test sensitivity and selectivity by using the same evaluation method in prior research focusing on classical inference (e.g., Woo et al., 2014; Eklund et al., 2016). Although we showed evaluation results of Bayesian inference in the present study, readers should be aware of this point while interpreting such results. In addition, future research should consider what will be better methods to compare sensitivity and selectivity between Bayesian and classical inference that are based on different statistical assumptions and frameworks. Fifth, because we only focused on SPM 12 to propose practical guidelines for end users, additional studies examining Bayesian inference implemented in other tools, such as FSL and AFNI (Woolrich, 2012; Webster, 2017), may also be required. Sixth, although we attempted to acquire as many image files containing results from first-order analyses for the replications from NeuroVault as possible, we could only download three datasets that contained data collected from 16 or more participants, which was the sample size of the moral psychology fMRI study. More image files might need to be shared through open fMRI data repositories by researchers to facilitate reanalyses of previous datasets with novel analysis methods, such as the Bayesian reanalyses performed in the present study, and, finally, to promote open science for better scientific practice.

Furthermore, related to updates on SPM 12 and fMRI analysis methods, future studies should address unanswered questions that could not be addressed in the present study due to its scope. First, although merely following default settings in SPM 12, a voxel size for normalization in particular, does not necessarily produce optimal outcomes as shown by recent research (Flandin and Friston, 2017; Mueller et al., 2017), we decided to follow such default settings due to the purpose and scope of the present study, suggesting how end users can implement a new analysis method, Bayesian second-level analysis, with currently and widely available tools and guidelines. Because we agree with the recent studies that SPM’s default settings should be carefully reconsidered, future studies should to test how Bayesian second-level analysis works with the recommended revised settings. Furthermore, based on findings from such future studies, guidelines for analysis for end users might also need to be revised and amended. Second, researchers have developed alternative methods for inference and thresholding, such as Statistical Non-parametric Mapping (SnPM; Nichols, 2012), Threshold-free Cluster Enhancement (TFCE; Smith and Nichols, 2009), and 3dClustSim with autocorrelation function (Cox et al., 2017); previous research has demonstrated that the application of the aforementioned methods can effectively address current issues on fMRI analysis, such as inflated false positives (Nichols and Holmes, 2002; Smith and Nichols, 2009; Eklund et al., 2016; Han and Glenn, 2017). However, because those methods are not available as basic functions in SPM 12, we did not test the alternative methods with Bayesian inference due to the limited scope of the present study, introducing Bayesian inference to end users who are familiar with SPM 12’s default settings. Hence, future studies should address such a limitation to examine and test Bayesian inference and the alternative analysis methods.

## Conclusion

In the present study, we compared outcomes from Bayesian and classical second-level analyses of first-level contrast images implemented in SPM 12. Although we only compared the simplest frequentist procedure, the one-group *t*-test, to the Bayesian counterpart due to the lack of available statistical and technical guidelines, we were able to show that Bayesian inference in the second-level fMRI analysis had practical and philosophical benefits. We also proposed practical guidelines for second-level Bayesian analysis in SPM 12: applying an effect size threshold of Cohen’s *D* = 0.5 (a medium effect size) and a Bayes factor threshold of logBF = 5 (very strong evidence). As Poldrack et al. (2017) argued, such a Bayesian approach will provide a more robust analysis methodology for fMRI studies with small, underpowered samples and will contribute to better scientific practice. We expect to have better ideas about how to utilize Bayesian inference, including both first- and second-level inference, for more-complex tests, such as two-group tests and linear regression, by conducting additional analyses in the future.

## Author Contributions

HH contributed to all stages of the research project and writing. JP contributed to the interpretation of findings, building theoretical framework, and writing.

## Conflict of Interest Statement

The authors declare that the research was conducted in the absence of any commercial or financial relationships that could be construed as a potential conflict of interest.

## Footnotes

1 http://www.neurovault.org/

2 As explained previously, the ES threshold value can be determined from a standard deviation value, which is identical to the square root of SPM.PPM.Cb containing the square of the conditional standard deviation of the prior parameter. In the case of the reanalysis of moral psychology fMRI data, the calculated SPM.PPM.Cb was 0.0027, and its square root was 0.0518. Thus, 0.0518 was the standard deviation value that represented Cohen’s *d* = 1.0, the default ES threshold value for the second-level analysis.

3 Because the calculated $\begin{array}{c} \sqrt{SPM.PPM.cB} \end{array}$, which represented the ES threshold value equivalent to Cohen’s *d* = 1.0, was 0.0518, the ES threshold value equivalent to Cohen’s *d* = 0.5 became $\frac{0.0518}{2}=0.0259$ = 0.0259.

## References

[B1] Amalric M. Dehaene S. (2016). Origins of the brain networks for advanced mathematics in expert mathematicians. *Proc. Natl. Acad. Sci.* 113 4909–4917. 10.1073/pnas.1603205113 27071124 PMC4983814

[B2] Ashburner J. Barnes G. Chen C.-C. Daunizeau J. Flandin G. Friston K. (2016). *SPM 12 Manual.* Available at: http://www.fil.ion.ucl.ac.uk/spm/doc/manual.pdf

[B3] Baker M. (2016). Is there a reproducibility crisis? *Nature* 533 452–454. 10.1038/533452a 27225100

[B4] Benjamini Y. Hochberg Y. (1995). Controlling the false discovery rate: a practical and powerful approach to multiple testing. *J. R. Stat. Soc. B* 57 289–300. 10.2307/2346101

[B5] Bennett C. M. Miller M. B. Wolford G. L. (2009). Neural correlates of interspecies perspective taking in the post-mortem Atlantic Salmon: an argument for multiple comparisons correction. *Neuroimage* 47:S125 10.1016/S1053-8119(09)71202-9

[B6] Button K. S. Ioannidis J. P. A. Mokrysz C. Nosek B. A. Flint J. Robinson E. S. J. (2013). Power failure: why small sample size undermines the reliability of neuroscience. *Nat. Rev. Neurosci.* 14 365–376. 10.1038/nrn3475 23571845

[B7] Chang C. Glover G. H. (2009). Relationship between respiration, end-tidal CO2, and BOLD signals in resting-state fMRI. *Neuroimage* 47 1381–1393. 10.1016/j.neuroimage.2009.04.048 19393322 PMC2721281

[B8] Cohen J. (1992). A power primer. *Psychol. Bull.* 112 155–159. 10.1037/0033-2909.112.1.155 19565683

[B9] Cohen J. (1994). The earth is round (*p* < 0.05): rejoinder. *Am. Psychol.* 50 1103–1103. 10.1037/0003-066X.50.12.1103

[B10] Cox R. W. Chen G. Glen D. R. Reynolds R. C. Taylor P. A. (2017). FMRI clustering in AFNI: false-Positive rates redux. *Brain Connect.* 7 152–171. 10.1089/brain.2016.0475 28398812 PMC5399747

[B11] Cui X. Li J. Song X. (2015). *Xjview.* Available at: http://www.alivelearn.net/xjview [accessed June 28 2015].

[B12] Eklund A. Nichols T. E. Knutsson H. (2016). Cluster failure: why fMRI inferences for spatial extent have inflated false-positive rates. *Proc. Natl. Acad. Sci. U.S.A.* 113 7900–7905. 10.1073/pnas.1602413113 27357684 PMC4948312

[B13] Ellison A. M. (1996). An introduction to Bayesian inference for ecological research and environmental decision-making. *Ecol. Appl.* 6 1036–1046. 10.2307/2269588

[B14] Eres R. Louis W. R. Molenberghs P. (2017). Common and distinct neural networks involved in fMRI studies investigating morality: an ALE meta-analysis. *Soc. Neurosci.* 1–15. 10.1080/17470919.2017.1357657 [Epub ahead of print]. 28724332

[B15] Flandin G. Friston K. J. (2017). Analysis of family-wise error rates in statistical parametric mapping using random field theory. *Hum. Brain Mapp.* 10.1002/hbm.23839 [Epub ahead of print]. 29091338 PMC6585687

[B16] Francis G. (2012). Too good to be true: publication bias in two prominent studies from experimental psychology. *Psychon. Bull. Rev.* 19 151–156. 10.3758/s13423-012-0227-9 22351589

[B17] Friston K. J. Harrison L. Penny W. (2003). Dynamic causal modelling. *Neuroimage* 19 1273–1302. 10.1016/S1053-8119(03)00202-7 12948688

[B18] Friston K. J. Penny W. (2003). Posterior probability maps and SPMs. *Neuroimage* 19 1240–1249. 10.1016/S1053-8119(03)00144-7 12880849

[B19] Gelman A. Hill J. Yajima M. (2012). Why we (Usually) don’t have to worry about multiple comparisons. *J. Res. Educ. Effs.* 5 189–211. 10.1080/19345747.2011.618213

[B20] Gigerenzer G. (2004). Mindless statistics. *J. Socio. Econ.* 33 587–606. 10.1016/j.socec.2004.09.033

[B21] Glover G. H. (2009). *SPIRAL IN/OUT Postprocessing for FMRI.* Available at: http://rsl.stanford.edu/glover/fmriutil/sprlio_postprocessing.pdf

[B22] Glover G. H. Law C. S. (2001). Spiral-in/out BOLD fMRI for increased SNR and reduced susceptibility artifacts. *Magn. Reson. Med.* 46 515–522. 10.1002/Mrm.1222 11550244

[B23] Glover G. H. Li T. Q. Ress D. (2000). Image-based method for retrospective correction of physiological motion effects in fMRI: RETROICOR. *Magn. Reson. Med.* 44 162–167. 10.1002/1522-2594(200007)44:1<162::AID-MRM23>3.0.CO;2-E 10893535

[B24] Gordon E. M. Laumann T. O. Gilmore A. W. Newbold D. J. Greene D. J. Berg J. J. (2017). Precision functional mapping of individual human brains. *Neuron* 95 791.e7–807.e7. 10.1016/j.neuron.2017.07.011 28757305 PMC5576360

[B25] Gorgolewski K. J. Varoquaux G. Rivera G. Schwarz Y. Ghosh S. S. Maumet C. (2015). NeuroVault.org: a web-based repository for collecting and sharing unthresholded statistical maps of the human brain. *Front. Neuroinform.* 9:8. 10.3389/fninf.2015.00008 25914639 PMC4392315

[B26] Greene J. D. Nystrom L. E. Engell A. D. Darley J. M. Cohen J. D. (2004). The neural bases of cognitive conflict and control in moral judgment. *Neuron* 44 389–400. 10.1016/j.neuron.2004.09.027 15473975

[B27] Greene J. D. Sommerville R. B. Nystrom L. E. Darley J. M. Cohen J. D. (2001). An fMRI investigation of emotional engagement in moral judgment. *Science* 293 2105–2108. 10.1126/science.1062872 11557895

[B28] Han H. (2017). Neural correlates of moral sensitivity and moral judgment associated with brain circuitries of selfhood: a meta-analysis. *J. Moral Educ.* 46 97–113. 10.1080/03057240.2016.1262834

[B29] Han H. Chen J. Jeong C. Glover G. H. (2016). Influence of the cortical midline structures on moral emotion and motivation in moral decision-making. *Behav. Brain Res.* 302 237–251. 10.1016/j.bbr.2016.01.001 26772629

[B30] Han H. Glenn A. L. (2017). Evaluating methods of correcting for multiple comparisons implemented in SPM12 in social neuroscience fMRI studies: an example from moral psychology. *Soc. Neurosci.* 1–11. 10.1080/17470919.2017.1324521 [Epub ahead of print]. 28446105

[B31] Head M. L. Holman L. Lanfear R. Kahn A. T. Jennions M. D. (2015). The extent and consequences of P-Hacking in science. *PLOS Biol.* 13:e1002106. 10.1371/journal.pbio.1002106 25768323 PMC4359000

[B32] Henson R. N. A. Price C. J. Rugg M. D. Turner R. Friston K. J. (2002). Detecting latency differences in event-related BOLD responses: application to words versus nonwords and initial versus repeated face presentations. *Neuroimage* 15 83–97. 10.1006/nimg.2001.0940 11771976

[B33] Ioannidis J. P. A. (2005). Why most published research findings are false. *PLOS Med.* 2:e124. 10.1371/journal.pmed.0020124 16060722 PMC1182327

[B34] Jeffreys H. (1961). *Theory of Probability.* Oxford: Oxford University Press.

[B35] Kass R. E. Raftery A. E. (1995). Bayes Factors. *J. Am. Stat. Assoc.* 90 773–795. 10.2307/2291091

[B36] Lieberman M. D. Cunningham W. A. (2009). Type I and Type II error concerns in fMRI research: re-balancing the scale. *Soc. Cogn. Affect. Neurosci.* 4 423–428. 10.1093/scan/nsp052 20035017 PMC2799956

[B37] Love J. Selker R. Marsman M. Jamil T. Dropmann D. Verhagen A. J. (2017). *JASP (Version 0.8.2).* Available at: https://jasp-stats.org/

[B38] Magerkurth J. Mancini L. Penny W. Flandin G. Ashburner J. Micallef C. (2015). Objective Bayesian fMRI analysis-a pilot study in different clinical environments. *Front. Neurosci.* 9:168. 10.3389/fnins.2015.00168 26029041 PMC4428130

[B39] Maxwell S. E. (2004). The persistence of underpowered studies in psychological research: causes, consequences, and remedies. *Psychol. Methods* 9 147–163. 10.1037/1082-989X.9.2.147 15137886

[B40] Mueller K. Lepsien J. Möller H. E. Lohmann G. (2017). Commentary: cluster failure: Why fMRI inferences for spatial extent have inflated false-positive rates. *Front. Hum. Neurosci.* 11:345. 10.3389/fnhum.2017.00345 28701944 PMC5487467

[B41] Neumann J. Lohmann G. (2003). Bayesian second-level analysis of functional magnetic resonance images. *Neuroimage* 20 1346–1355. 10.1016/S1053-8119(03)00443-9 14568503

[B42] Nichols T. (2012). Multiple testing corrections, nonparametric methods, and random field theory. *Neuroimage* 62 811–815. 10.1016/j.neuroimage.2012.04.014 22521256

[B43] Nichols T. Hayasaka S. (2003). Controlling the familywise error rate in functional neuroimaging: a comparative review. *Stat. Methods Med. Res.* 12 419–446. 10.1191/0962280203sm341ra 14599004

[B44] Nichols T. Holmes A. P. (2002). Nonparametric permutation tests for functional neuroimaging: a primer with examples. *Hum. Brain Mapp.* 15 1–25. 10.1002/hbm.1058 11747097 PMC6871862

[B45] Nuzzo R. (2014). Scientific method: statistical errors. *Nature* 506 150–152. 10.1038/506150a 24522584

[B46] Open Science Collaboration. (2015). Estimating the reproducibility of psychological science. *Science* 349:aac4716. 10.1126/science.aac4716 26315443

[B47] Pashler H. Wagenmakers E. J. (2012). Editors’ introduction to the special section on replicability in psychological science: a crisis of confidence? *Perspect. Psychol. Sci*. 7 528–530. 10.1177/1745691612465253 26168108

[B48] Peng R. (2015). The reproducibility crisis in science: a statistical counterattack. *Significance* 12 30–32. 10.1111/j.1740-9713.2015.00827.x

[B49] Penny W. (2005). *Bayesian Analysis of Single-Subject fMRI Data: User Guide.* Available at: www.fil.ion.ucl.ac.uk/~wpenny/bayes-fmri/user_guide.pdf

[B50] Penny W. Flandin G. (2005). “Bayesian analysis of fMRI data with spatial priors,” in *Proceedings of the Joint Statistical Meeting (JSM) American Statistical Association* (Boston, MA).

[B51] Penny W. Friston K. (2007). “Bayesian treatments of neuroimaging data,” in *Bayesian Brain: Probabilistic Approaches to Neural Coding* eds Doya K. Ishii S. Pouget A. Rao R. P. N. (Cambridge, MA: MIT Press) 91–108.

[B52] Poldrack R. A. Baker C. I. Durnez J. Gorgolewski K. J. Matthews P. M. Munafò M. R. (2017). Scanning the horizon: towards transparent and reproducible neuroimaging research. *Nat. Rev. Neurosci.* 18 115–126. 10.1038/nrn.2016.167 28053326 PMC6910649

[B53] Rouder J. N. Speckman P. L. Sun D. Morey R. D. Iverson G. (2009). Bayesian t tests for accepting and rejecting the null hypothesis. *Psychon. Bull. Rev.* 16 225–237. 10.3758/PBR.16.2.225 19293088

[B54] Scott J. G. Berger J. O. (2010). Bayes and empirical-Bayes multiplicity adjustment in the variable-selection problem. *Ann. Stat.* 38 2587–2619. 10.1214/10-AOS792

[B55] Sidén P. Eklund A. Bolin D. Villani M. (2017). Fast Bayesian whole-brain fMRI analysis with spatial 3D priors. *Neuroimage* 146 211–225. 10.1016/j.neuroimage.2016.11.040 27876654

[B56] Simmons J. P. Nelson L. D. Simonsohn U. (2011). False-Positive psychology. *Psychol. Sci.* 22 1359–1366. 10.1177/0956797611417632 22006061

[B57] Smith S. M. Nichols T. E. (2009). Threshold-free cluster enhancement: addressing problems of smoothing, threshold dependence and localisation in cluster inference. *Neuroimage* 44 83–98. 10.1016/j.neuroimage.2008.03.061 18501637

[B58] Stern H. S. (2016). A test by any other name: P values, bayes factors, and statistical inference. *Multivariate Behav. Res.* 51 23–29. 10.1080/00273171.2015.1099032 26881954 PMC4809350

[B59] Trafimow D. Marks M. (2015). Editorial. *Basic Appl. Soc. Psych.* 37 1–2. 10.1080/01973533.2015.1012991

[B60] Wagenmakers E.-J. (2007). A practical solution to the pervasive problems ofp values. *Psychon. Bull. Rev.* 14 779–804. 10.3758/BF03194105 18087943

[B61] Wagenmakers E.-J. Marsman M. Jamil T. Ly A. Verhagen J. Love J. (2017). Bayesian inference for psychology. Part I: theoretical advantages and practical ramifications. *Psychon. Bull. Rev.* 10.3758/s13423-017-1343-3 [Epub ahead of print]. 28779455 PMC5862936

[B62] Wasserstein R. L. Lazar N. A. (2016). The ASA’s statement on *p*-values: context, process,∖r∖nand purpose. *Am. Stat* 70 129–133. 10.1080/00031305.2016.1154108

[B63] Webster M. A. (2017). *FEAT/UserGuide.* Available at: https://fsl.fmrib.ox.ac.uk/fsl/fslwiki/FEAT/UserGuide

[B64] Woo C.-W. Krishnan A. Wager T. D. (2014). Cluster-extent based thresholding in fMRI analyses: pitfalls and recommendations. *Neuroimage* 91 412–419. 10.1016/j.neuroimage.2013.12.058 24412399 PMC4214144

[B65] Woolrich M. W. (2012). Bayesian inference in FMRI. *Neuroimage* 62 801–810. 10.1016/j.neuroimage.2011.10.047 22063092

