# Supplementary material for: Using SPM 12’s Second-Level Bayesian Inference Procedure for fMRI Analysis: Practical Guidelines for End Users
Source: Front Neuroinform. 2018 Feb 2;12:1. doi: 10.3389/fninf.2018.00001 (PMC5801291; doi:10.3389/fninf.2018.00001)
Supplement: Supplementary file 1 [file Table_1.PDF]

Supplemental Materials

Table S1

The order of first-level contrast images entered to the second-level analysis model for each trial

Sample size	Trial number									
	1	2	3	4	5	6	7	8	9	10
1	1	12	1	15	11	1	12	3	7	4
2	2	6	2	8	3	4	13	9	12	8
3	3	5	8	1	1	16	6	11	5	13
4	4	1	4	16	9	6	11	6	15	5
5	5	14	5	6	15	14	1	4	14	6
6	6	11	11	13	2	5	14	8	10	1
7	7	3	14	12	7	15	15	2	16	15
8	8	16	15	2	10	12	2	16	3	10
9	9	10	13	14	8	7	4	7	11	16
10	10	9	6	7	6	3	16	15	4	14
11	11	7	16	10	14	10	7	14	6	11
12	12	15	10	3	4	9	3	12	2	3
13	13	2	3	4	13	11	5	1	8	2
14	14	13	7	11	5	2	8	10	1	12
15	15	4	9	9	12	13	9	13	13	7
16	16	8	12	5	16	8	10	5	9	9

Note . Each number represents the numbered file name of each first-level contrast image stored in NeuroVault.
